# Impact of the Antioxidant Hydroxytyrosol on the Quality of Post-Thawed Stallion Semen

**DOI:** 10.1155/2024/6558480

**Published:** 2024-04-30

**Authors:** Yousef M. Alharbi, Mohamed Ali, Mohammed S. Alharbi

**Affiliations:** ^1^Department of Veterinary Medicine, College of Veterinary Medicine, Qassim University, Buraydah 51452, Saudi Arabia; ^2^Department of Animal Production and Food, College of Agriculture and Veterinary Medicine, Qassim University, Buraydah 51452, Saudi Arabia

## Abstract

The objective of this study was to investigate the impact of including hydroxytyrosol (HT) antioxidant on the viability of sperm after the processes of cooling and freezing. HT antioxidants were used in the HF-20 extender at concentrations of 1.25, 2.5, 5, and 10 *μ*g/ml. The HF-20 extender was a basic extender and was used for the control group. The post-thawed semen exhibited significantly higher total motility in the 2.5 HT and 10 HT treatment groups than the control group. The straight line velocity (VSL) of the 2.5 HT group exhibited a significantly high value compared with the control group. However, the average path velocity (VAP), linearity (LIN), straightness index (STR), and wobble (WOB) revealed identical findings across all groups. The findings of the analysis of HOST, normal morphology, major abnormalities, and minor abnormalities revealed that there were no significant differences between the HT groups and the control groups. Nevertheless, the use of HT antioxidant for freezing semen led to a notable enhancement (*p* < 0.05) in both acrosome integrity and vitality tests when compared to the control group. In this case, the lower quantities of HT (1.25 and 2.5 *μ*g/ml; *p* < 0.05) preserve the DNA fragmentation compared with the 5 HT, 10 HT, and control groups. In conclusion, the HT antioxidant has shown the capacity to enhance the quality of frozen-thawed spermatozoa and positively influence the viability and integrity of DNA inside the frozen-thawed spermatozoa. Additional research should be conducted to assess the fertility potential of cryopreserved stallion semen.

## 1. Introduction

A high rate of pregnancy in horses is influenced by two primary factors: mare fertility and reproductive management. However, to enhance the effectiveness of artificial insemination (AI), it is essential to use good-quality semen. The objective of artificial insemination is to introduce a sufficient quantity of viable sperm into the female reproductive system at the optimal period. This procedure may be conducted with fresh, cooled, or cryopreserved semen. Spermatozoa may be retained and transported at a temperature range of 5–8°C for 12–36 hours, but spermatozoa frozen within a temperature range of −196°C can be stored in liquid nitrogen for extended periods of time. The concentration of semen per milliliter has a direct impact on the quality of preserved semen. Consequently, it is necessary to dilute extremely concentrated semen to achieve the appropriate concentration prior to use [1]. According to Aurich et al. [1], it is recommended that the minimum sperm concentration for artificial insemination (AI) using frozen semen should be at least 250 × 10^6^ progressive motility sperm (PMS). The effectiveness of artificial insemination in horses is contingent upon two crucial factors: sperm quality and uterine deposit at the appropriate moment for insemination [2]. According to Kowalczyk et al. [2], a mare's likelihood of achieving a successful pregnancy is increased when it is inseminated with frozen semen within a time frame of 4–6 hours before or after ovulation.

The process of cryopreservation and thawing exposes spermatozoa to a range of stressors, which may ultimately result in the decline of their ability to fertilise. Consequently, the procedure of cryopreservation has undergone many enhancements [3]. The adverse impact on stallion spermatozoa caused by oxidative stress (OS), which arises from an imbalance between reactive oxygen species (ROS) and antioxidants, leads to a considerable decline in their functionality. During the process of sperm cryopreservation and thawing, it has been shown that there is an elevation in the formation of ROS and a reduction in antioxidant levels [4, 5]. Hence, the operating system does indeed have an impact on the injuries sustained by spermatozoa throughout the process of cryopreservation. Consequently, the use of antioxidants that mitigate the impact of ROS may be beneficial in the prevention of cryoinjury generated by OS.

In recent times, there has been significant research conducted on natural antioxidants due to their exceptional ability to counteract oxidative stress [6, 7]. Hydroxytyrosol (HT) is a naturally occurring phenolic compound ([2-(3,4-dihydroxyphenyl) ethanol]) that exhibits potent scavengers of hydroxyl radicals (OH^*∗*^), peroxynitrite (ONOOH), and superoxide radicals (O(2)^*∗*^(−)), specifically known as a ROS scavenger. Numerous studies have shown the ability of HT to significantly reduce the buildup of ROS in diverse cellular contexts [7]. For example, it has been shown in many studies that HT has the ability to shield vascular endothelial cells from the harmful impacts of hydrogen peroxide [8, 9]. Additionally, HT has also been found to provide protection to mammary cells [10] and human peripheral blood mononuclear cells (PBMCs) [11]. To our knowledge, this work represents the first investigation into the potential of HT as an antioxidant in the cryopreservation process of stallion sperm. Previous research has examined the impact of antioxidants, such as HT, on the quality of sperm in many animal species, including the semen of bulls [12], rams [13], and goats [14]. Hence, the objectives of this research were to assess the impact of HT antioxidant supplementation to the basic extender (HF-20) on cooled and frozen-thawed sperm parameters of Arabian horses.

## 2. Materials and Methods

### 2.1. Animals and Extender Preparation

Four Arabian stallions were utilised, ranging in age from 4 to 10 years, and were chosen for inclusion in this research after a comprehensive assessment of their reproductive soundness. Each animal was kept in separate enclosures and provided with concentrated clover hay, fresh water, and integrated mineral licks. A rhythmical semen collection protocol was implemented, whereby estrus mares were subjected to collection twice a week per stallion.

The centrifuged media consisted of 6.0 gram (gm) of glucose, 0.37 gm of ethylene-diamine-tetra-acetic acid (EDTA), 0.37 gm of sodium citrate, 0.20 gm of sodium bicarbonate, 0.08 gm of streptomycin, and 100,000 international units (IUs) of penicillin, all dissolved in 100 ml of distilled water. A freezing extender HF-20 containing egg yolk was prepared using the following components: 5.0 gm of glucose, 0.3 gm of lactose, 0.3 gm of raffinose, 0.15 gm of sodium citrate, 0.05 gm of sodium phosphate, 0.05 gm of sodium potassium tartrate, and 3 ml of glycerol. These components were dissolved in 50 ml of distilled water. Subsequently, 8 ml of egg yolk (EY) was added as a basic buffer, and distilled water was added to reach a final volume of 100 ml. The HF-20 extender was enhanced with hydroxytyrosol at several concentrations of 0 (control), 1.25, 2.5, 5, and 10 *μ*g/ml. The pH of the extender was adjusted using a sodium bicarbonate buffer. The pH measurement was calibrated via a pH meter (Hanna, Model HI-2212; Woonsocket, RI, USA).

### 2.2. Collecting and Processing of Semen

In this experiment, the estrus mare was used as a means for semen collection. Following the process of semen collection, the gel component was promptly extracted from the semen sample. The specimen was obtained via aseptic gauze and then transported to a thermostatically controlled water bath set at a temperature of 37°C. The volume of semen was quantified using a graduated cylinder. Subsequently, the ejaculate was assessed in terms of overall progressive motility (PM) and sperm concentration. The CASA system (ISAS program, Prosser R + D; Paterna, Valencia, Spain) was used to assess sperm concentration and motility. This research used samples that had a minimum concentration of 200 × 10^6^ sperm/ml and showed a total motility above 60%. Each ejaculate's filtered semen was diluted in a 1 : 1 ratio with centrifuged media and then separated into five equal portions. The aliquots underwent centrifugation at a force of 900 g for 10 minutes. Subsequently, the seminal plasma was separated and discarded, while each individual sample was then resuspended using FH-20 that had been supplemented with varying concentrations of HT.

The final concentration of semen after dilution was determined to be 200 × 10^6^ sperm/ml. Before the assessment, each tube was subjected to a chilling procedure, reducing the temperature to 4°C, which lasted for 90 minutes. After that, the tubes were analyzed to assess the motility, morphology, acrosome integrity, and plasma membrane of the sperm. The cooled semen was filled into straws manually with a capacity of 0.5 ml. During the freezing process, the straws were placed horizontally on the surface of liquid nitrogen, with a distance between them of 9 cm, for 9 minutes according to the method described by Squires et al. [15]. Following that, they were expeditiously submerged in liquid nitrogen. The straws were collected and then inserted into goblets, which were then transferred to a tank filled with liquid nitrogen for storage.

### 2.3. The Assessment of Frozen Semen Quality

The frozen straws underwent a thawing process by being immersed in a water bath at a temperature of 38°C for 60 seconds. Subsequently, the contents of the straws were evacuated into a tiny tube that had been warmed. The ISAS program (the CASA system) was used to test the general and progressive motility of the samples. Spermatozoa with VAP values <10 *μ*m/s were considered immotile. However, spermatozoa with VAP >20 *μ*m/s, VSL >30 *μ*m/s, and VCL >45 *μ*m/s were considered motile [16]. The evaluation included the examination of plasma membrane integrity, morphological defects, acrosome integrity, viability, and DNA fragmentation. The examination of frozen semen was carried out at the AI Centre for Animal Production and Breeding, affiliated with Qassim University.

### 2.4. The Evaluation of Sperm Motility

The ISAS program was used to evaluate the motility patterns of semen immediately upon dilution or post-thawed semen. A small portion (2.7 *μ*L) from each sample was applied onto a prewarmed counting chamber (ISAS disposable slide), and the motility of the semen was evaluated using five digital photographs captured from various fields. This assessment was conducted using *a* ×10 negative-phase contrast objective and a warm stage set at a temperature of 38°C. The study assessed motility patterns using several parameters, including total motile sperm (TMS (%)), progressive motile (PM (%)), curvilinear speed (VCL (*μ*m/s)), rectilinear speed (VSL (*μ*m/s)), average value (VAP (*μ*m/s)), linearity index (LIN (%)), straightness index (STR (%)), and wobble (WOB (%)). A minimum of 300 spermatozoa were examined in each sample, and the visual representations were interpreted within a duration of one second.

### 2.5. Plasma Membrane Integrity

A hypo-osmotic swelling test (HOST) was used to evaluate the integrity of the spermatozoa's plasma membrane. The presence of a coiled tail was examined in a total of at least 100 sperm cells for each sample. This assessment was conducted using phase contrast microscopy at a magnification of 400 times. In the study conducted by Neild et al. [17], an experiment was performed whereby a solution containing glucose with an osmolality of 100 mOsmol was combined with 20 *μ*L of semen. This combination was then subjected to incubation at a temperature of 37°C for 50 minutes under a water bath.

### 2.6. Vitality Test

The assessment of sperm viability was performed using a kit supplied by Halotech DNA S.L., a Madrid-based firm in Spain. The kits include acridine orange (AO) and propidium iodide (PI). AO is a cell-permeant DNA-binding dye that stains both live and dead cells with green fluorescence, while PI is a membrane-impermeant DNA-binding dye that stains dead cells red with damaged membranes. At the outset, the frozen was diluted to a concentration of 10–15 × 10^6^ sperm/ml. Following this, a quantity of 10 *μ*L of cryopreserved semen was mixed with a solution containing 1.0 *μ*L of AO and PI. Subsequently, the combination was examined using a fluorescent microscope produced by Optika Srl, Italy. The live sperm had green fluorescence as a consequence of AO retention, but the damaged sperm exhibited red fluorescence owing to PI penetration. In each case, an evaluation was conducted on a sample size of 300 spermatozoa.

### 2.7. Examination of DNA Fragmentation

The Halomax kit, which was created by Halotech DNA S.L. in Spain, was used for the assessment of DNA fragmentation in equines. The Sperm-Halomax® (Halotech DNA S.L., Madrid, Spain) methodology is based on the sperm chromatin dispersion test (SCDt) as outlined in the study conducted by Cortés-Gutiérrez et al. [18]. The initial volume of sperm was diluted in agarose to get a final concentration of 1 × 10^6^ sperm/ml. The diluted sample was next moved to racks, where it underwent lysis. Following this, the sample was stained with propidium iodide to produce slides for examination. The investigation of DNA fragmentation was conducted using a fluorescence microscope (Optika Srl, Italy) at a magnification of 1000x. To determine the sperm DNA fragmentation index, a count of 300 sperm was conducted on each slide. The distinguishing feature of this index is the occurrence of a halo around the head of the sperm, resulting from the dispersion of chromatin inside agarose. The presence of a halo signifies the presence of DNA damage, which may manifest as either single-strand breaks or double-strand breaks. The dispersion of DNA fragments from the sperm head is observed, with larger chromatin fragments displaying relatively less displacement than smaller pieces, leading to the creation of a distinct “halo” around the sperm head. The phenomenon of sperm fragmentation is considered to occur when the diameter of the halo exceeds twice the radius of the sperm head.

### 2.8. Morphology

The examination of sperm morphology will be conducted with Hancock's solution. A 15 *μ*L aliquot of semen was combined with an equal volume of a sterile and heated solution in a clean and warm tube. The solution was carefully deposited, concealed, and then examined using oil immersion at a magnification of 1000 times. The morphological defects of spermatozoa were categorized as sperm abnormalities based on their impact on fertility. Major defects encompassed a majority of abnormalities observed in the head and midpiece, as well as proximal cytoplasmic droplets and single abnormalities occurring at a high frequency. However, minor defects included looped tails, detached sperm heads, and distal cytoplasmic droplets [19].

### 2.9. Acrosome Integrity

A single droplet of semen sample was combined with an equivalent droplet of isotonic Hancock's solution containing 6% formaldehyde on a microscope slide that had been cleaned and heated. Subsequently, the combination was appropriately shielded and subjected to examination using a light microscope, specifically at a magnification level of ×1000. A minimum of 200 spermatozoa were meticulously observed to identify any potential abnormalities in the acrosome.

### 2.10. Statistical Analysis

Descriptive analyses were used to evaluate a range of variables, such as TM (total motility), PM (progressive motility), VCL (curvilinear velocity), VSL (straight line velocity), VAP (average path velocity), STR (straightness), LIN (linearity), WOB (wobble), acrosome integrity, vitality, HOST (hypo-osmotic swelling test), morphological defect, vitality test, and DNA fragmentation. The data were presented in the form of means and the standard error of the mean (SEM). The statistical comparisons between groups were performed using a one-way analysis of variance (ANOVA) with a significance threshold of *p* < 0.05, using SPSS version 21.

## 3. Result

The motility and speed characteristics, acrosome integrity, HOST, and defect of the morphology of stallion sperm after cooled storage are represented in Table 1. The control group and the extenders in 1.25, 2.5, and 5 HT groups exhibited a statistically significant higher total motility than the 10 HT group. There was no significant difference among groups in the PM and sperm kinematic values such as VCL, VSL, VAP, STR, LIN, and WOB. The prevalence of major abnormalities was significantly higher in the control group than in the HT group. However, there was no significant difference in the normal morphology of sperm and minor abnormalities. In addition, there was no significant difference in the proportion of acrosome integrity and HOST among the various groups.

Table 2 shows the motility and kinematic parameters, acrosome integrity, HOST, and defect of the morphology of stallion sperm after cryopreservation. The extender in the 2.5 HT and 10 HT groups had a statistically significant higher total motility than the control group. The progressive motility values exhibited no significant differences among the various extenders. There were no statistically significant differences across groups in terms of the speed characteristics such as VCL, VAP, LIN, STR, and WOB of stallion sperm. However, a significant difference in VSL was observed, with the 5 HT group exhibiting significantly higher levels than the control group. The proportions of minor and major abnormalities, as well as the normal morphology of sperm, were found to be similar across all groups. Likewise, no statistically significant difference in HOST test scores was found across the groups. The acrosome integrity of frozen-thawed semen exhibited a statistically significant increase in the 1.25 HT, 2.5 HT, 5 HT, and 10 HT groups as compared with the control group.

**Figure 1:**
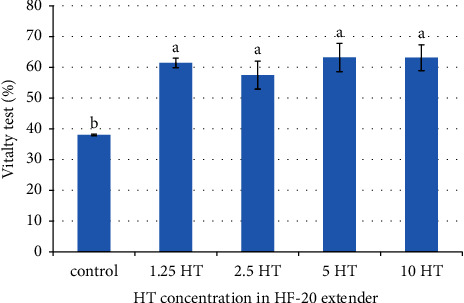
Effect of different concentrations of HT on the vitality test of post-thawed stallion semen. ^a,b^Values showed a significant difference at *p* < 0.05.

**Figure 2:**
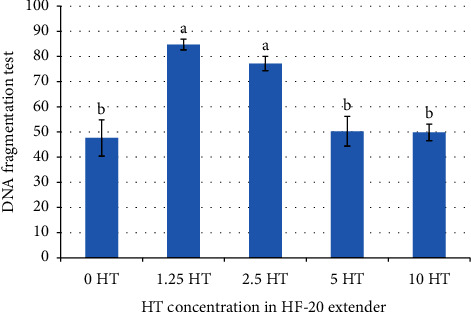
Effect of different concentrations of HT on DNA intact of post-thawed stallion semen. ^a,b^Values showed a significant difference at *p* < 0.05.

Figure 1 displays the results of the percentages of sperm vitality in the experiment, which were 61.42 ± 1.56, 57.45 ± 4.56, 63.20 ± 4.60, and 63.14 ± 4.18% in extenders supplemented with 1.25, 2.5, 5, and 10 *μ*g/ml of HT, respectively. These values were found to be significantly higher (*p* < 0.05) than the control group, which had a sperm viability of 38.04 ± 0.25%. A statistically significant effect (*p* < 0.05) was observed for intact DNA in extenders supplemented with low concentrations of HT (1.25 and 2.5 *μ*g/ml; 84.76 ± 2.12, 77.18 ± 2.83%, respectively) compared with the control, 5 HT, and 10 HT groups (50.26 ± 5.91, 49.79 ± 3.28, and 47.63 ± 7.19%, respectively) (Figure 2).

## 4. Discussion

The cryopreservation of equine sperm provides several advantages for horse breeders in comparison with the storage of fresh sperm. Nevertheless, recent research has shown that the levels of ROS experience a notable rise throughout the process of cryopreservation, leading to the disruption of sperm characteristics and subsequent fertilisation outcomes [20]. Consequently, the presence of elevated levels of ROS results in heightened oxidative stress, leading to lipid peroxidation. This process, in turn, impacts the structure of cellular membranes and disrupts their various functions, including membrane fluidity, membrane enzymes, ionic gradients, transport receptors, and transport processes [20, 21]. In the present study, the incorporation of antioxidants into semen extenders shows promise in counteracting the adverse impact of oxidative stress. Antioxidants possess the ability to capture free radicals and subsequently terminate the chain reaction, thereby preserving the redox state and compensating for their capacity to diminish molecular oxygen.

The findings of this study indicated that the addition of 2.5 and 10 *μ*g/mL of HT to extenders resulted in a 10% increase in total motility compared with the control treatment. These results are consistent with the findings of Krishnappa et al. [22], who observed an enhancement in total motility with the supplementation of 80 mM HT to ram sperm. The findings of this study indicate that the presence of HT may mitigate the concentration of ROS, thereby minimising the adverse impact of moderately high ROS levels on sperm motility. This effect is mostly achieved by the depletion of intracellular ATP and a subsequent decrease in the phosphorylation of axonal proteins [23]. Nevertheless, as a result of the supplementation of HT in extenders, TM of sperm exhibited a statistically significant increase when compared to the control group. Furthermore, there was a slight increase in PM in the experimental group as compared to the control group. There was no significant difference (*p* > 0.05) in the characteristics of velocity and relative velocity (VCL, VSL, VAP, LIN, and STR) among groups. This phenomenon, which has a resemblance to a previously observed pattern, has been documented in goats [14] and rams [22]. Nevertheless, the use of 5 *μ*g/ml of HT has the potential to enhance the value of VSL. According to Hirata et al. [24], mitochondria serve as the primary location for the generation of adenosine triphosphate (ATP), a process that occurs via the utilisation of the electron transport chain and oxidative phosphorylation, in response to energy requirements. Nevertheless, research by Arando et al. [25] conducted on animals examined the effects of HT in extenders on mitochondrial membrane potential in post-thawed sperm, and the results indicated that there was no improvement found in comparison with the control group. Hence, it can be shown that there was no significant increase in the energy requirements of the sperm. Conversely, it was reported that hydroxytyrosol enhanced the mitochondrial electron transport chain and was involved in mitochondrial integrity in rats [26]. It indicated the velocity and relative velocity attributes exhibited by stallion sperm after the freezing process.

According to Dutta et al. [27], an elevated concentration of polyunsaturated fatty acids in sperm membranes has the potential to interact with ROS, therefore influencing the fluidity of the membrane. This interaction may also facilitate the entry of calcium ions (Ca2+), leading to the reorganisation of membrane proteins and the destabilisation of the plasma membrane. Based on the present findings, the supplementation of HT at several doses ranging from 5 to 10 *μ*g/ml has shown potential for enhancing membrane integrity. However, it is noteworthy that these improvements were not found to be statistically significant when compared to the control treatment. Therefore, there is no effect of adding HT on the integrity of the plasma sperm membrane. In contrast, previous studies have shown an observed enhancement in membrane integrity with the use of alternative antioxidants within the extender media [28, 29].

The acrosome is a distinct anatomical feature of sperm that consists of membranes and proteins, rendering it particularly susceptible to damage caused by ROS [27]. In this particular setting, the supplementation of extenders with varying doses of HT (ranging from 1.25 to 10 *μ*g/ml) had a beneficial impact on the integrity of the acrosome. The findings of this study align with other studies conducted on goat semen [13] and ram semen [22].

According to research conducted by Grasso et al. [30], the inclusion of HT may have a beneficial effect on preventing DNA damage. This study observed a substantial increase of 19% in the preservation of intact DNA in both the 1.25 HT and 2.5 HT groups, as compared to the control group. According to research conducted by Warleta et al. [10], the inclusion of HT in oil-derived antioxidants was shown to enhance the safeguarding effect against oxidative DNA damage in mammary cells. Similarly, another study by Ilavarasi et al. [11] observed that the addition of HT increased the protection against oxidative DNA damage in human peripheral blood mononuclear cells (PBMCs).

Hydroxytyrosol, a polyphenol, has attracted broad interest due to its strong antioxidant, anti-inflammatory, and antibacterial activities [31]. Hydroxytyrosol showed antioxidant properties by scavenging ROS, reducing the accumulation of ROS, and promoting the expression of antioxidant enzymes via activating nuclear factor E2-related factor 2 (Nrf2) signaling [31]. Moreover, hydroxytyrosol enhanced the activity of antioxidant enzymes (CAT, T-AOC, SOD, and GSH-PX), which led to reduced lipid peroxidation (MDA) in pig semen stored at 17°C [32] and catalyzed the decomposition of hydrogen peroxide (H_2_O_2_) in postweaning piglets [33]. Furthermore, Kedechi et al. [34] reported that the addition of hydroxytyrosol reduced sperm DNA oxidation in human semen. It is hypothesized that the addition of HT could improve the quality parameters and viability of stallion sperm during cryopreservation, which is crucial for improving the ability of postfreezing sperm to fertilise. The present study elucidated that 1.25 and 2.5 *μ*g/ml of HT are eligible to enhance sperm motility and viability postfreezing.

However, elevated levels of these antioxidants may have detrimental effects on sperm. There are a lot of data indicating that increased concentrations may not always result in improved quality, but rather may have detrimental effects [14]. Therefore, it might be suggested that the HT inhibited H_2_O_2_ and decreased its effect on DNA at lower concentrations. H_2_O_2_ passes through a membrane and acts directly on the DNA. However, at higher concentrations, the HT effect was deleterious, because antioxidants can become oxidants to cells if they exceed.

## 5. Conclusion

Hydroxytyrosol can be recommended as a good antioxidant in stallion semen extenders. The concentrations of 1.25 and 2.5 *μ*g/ml could be used for the cryopreservation of Arabic stallion semen and increased the viability and DNA intact of post-thawed semen compared with the control group. Additionally, in vivo work is needed using HT for artificial insemination in mares to determine its effect on fertility rates.

## Figures and Tables

**Table 1:** The effect of adding HT at different concentrations on motility, acrosome integrity, HOST, and defect of the morphology in cooled stallion semen.

Parameters	Control	Hydroxytyrosol (*μ*g/ml)			
		1.25	2.50	5.00	10.00
TM (%)	80.71 ± 3.84^a^	78.33 ± 4.40^a^	81.00 ± 3.31^a^	80.00 ± 3.65^a^	65.00 ± 2.88^b^
PM (%)	25.66 ± 5.35	29.50 ± 7.13	17.06 ± 3.74	17.05 ± 5.30	12.25 ± 4.95
VCL (*μ*m/s)	90.27 ± 1.41	91.93 ± 0.74	91.13 ± 0.89	89.85 ± 0.77	91.12 ± 0.02
VSL (*μ*m/s)	23.17 ± 0.67	25.03 ± 0.83	24.52 ± 0.70	24.85 ± 0.95	24.10 ± 0.40
VAP (*μ*m/s)	46.10 ± 0.76	46.56 ± 0.66	46.17 ± 0.94	46.47 ± 0.79	46.25 ± 0.75
LIN (%)	27.31 ± 0.64	27.65 ± 1.08	27.18 ± 0.72	28.27 ± 1.03	26.85 ± 0.25
STR (%)	53.14 ± 1.00	53.80 ± 1.49	52.94 ± 0.92	53.60 ± 1.26	52.40 ± 1.80
WOB (%)	52.30 ± 0.45	52.40 ± 1.27	51.33 ± 0.78	52.44 ± 0.92	51.27 ± 1.27
Normal morphology (%)	83.77 ± 1.41	81.43 ± 1.84	78.36 ± 2.94	78.39 ± 2.47	79.10 ± 2.09
Major abnormalities (%)	9.99 ± 1.58^a^	7.89 ± 1.19^b^	8.70 ± 2.14^b^	9.11 ± 1.84^b^	6.80 ± 1.69^b^
Minor abnormalities (%)	6.97 ± 2.03	10.65 ± 1.58	12.93 ± 2.73	12.49 ± 2.49	14.09 ± 2.16
HOST (%)	74.96 ± 3.45	73.00 ± 2.20	69.30 ± 4.41	70.10 ± 3.22	66.39 ± 4.36
Acrosome integrity (%)	89.03 ± 1.28	92.46 ± 1.43	90.60 ± 1.98	90.63 ± 1.90	90.86 ± 0.13

^a,b,ab^Values with different superscripts in the same row among groups showed significant differences at *p* < 0.05, ANOVA.

**Table 2:** The effect of adding HT at different concentrations on acrosome integrity, plasma membrane integrity, and morphology in postthawed-frozen stallion semen.

Parameters	Control	Hydroxytyrosol (*μ*g/1 ml)			
		1.25	2.50	5.00	10.00
TM (%)	37.73 ± 1.56^b^	40.71 ± 2.54^ab^	47.14 ± 3.59^a^	44.29 ± 3.68^ab^	55.00 ± 6.45^a^
PM (%)	15.35 ± 3.88	18.27 ± 3.68	18.90 ± 1.76	16.75 ± 1.14	17.75 ± 1.55
VCL (*μ*m/s)	75.05 ± 7.00	83.62 ± 1.71	85.22 ± 1.04	83.16 ± 2.89	87.95 ± 1.15
VSL (*μ*m/s)	21.45 ± 1.59^b^	24.65 ± 1.47^ab^	24.21 ± 1.03^ab^	30.56 ± 6.50^a^	23.62 ± 0.17^ab^
VAP (*μ*m/s)	39.37 ± 2.55	41.82 ± 0.71	43.95 ± 1.03	43.77 ± 1.27	44.25 ± 0.35
LIN (%)	30.01 ± 1.35	30.21 ± 2.43	29.05 ± 0.99	28.68 ± 0.83	27.37 ± 0.47
STR (%)	54.87 ± 1.22	59.18 ± 4.22	55.18 ± 1.32	59.01 ± 4.30	53.62 ± 0.67
WOB (%)	54.53 ± 2.36	51.05 ± 0.59	52.45 ± 0.58	51.52 ± 0.81	51.02 ± 0.22
Normal morphology (%)	77.76 ± 4.62	73.36 ± 3.89	73.85 ± 4.70	73.54 ± 2.34	73.12 ± 5.08
Major abnormalities (%)	7.58 ± 1.98	9.80 ± 1.66	7.11 ± 1.07	8.51 ± 0.93	6.51 ± 0.15
Minor abnormalities (%)	14.77 ± 3.41	16.83 ± 2.27	19.08 ± 3.61	17.94 ± 2.86	20.36 ± 4.95
HOST (%)	58.16 ± 4.04	60.56 ± 3.31	58.91 ± 4.14	65.56 ± 4.32	68.13 ± 4.83
Acrosome integrity (%)	77.98 ± 15.16^b^	90.78 ± 2.25^a^	92.42 ± 0.94^a^	91.51 ± 0.85^a^	92.93 ± 1.04^a^

^a,b,ab^Values with different superscripts in the same row among groups showed significant differences at *p* < 0.05, ANOVA.

## Data Availability

The data supporting the conclusions of this research can be obtained from the corresponding author upon a reasonable request.
